# ﻿A new jewel-like species of the pill-millipede genus *Sphaerobelum* Verhoeff, 1924 (Diplopoda, Sphaerotheriida, Zephroniidae) from Thailand

**DOI:** 10.3897/zookeys.1181.109076

**Published:** 2023-09-29

**Authors:** Ruttapon Srisonchai, Natdanai Likhitrakarn, Chirasak Sutcharit, Thierry Backeljau, Piyatida Pimvichai

**Affiliations:** 1 Department of Biology, Faculty of Science, Khon Kaen University, Khon Kaen 40002, Thailand Khon Kaen University Khon Kaen Thailand; 2 Program of Agriculture, Faculty of Agricultural Production, Maejo University, Chiang Mai 50290, Thailand Maejo University Chiang Mai Thailand; 3 Animal Systematics Research Unit, Department of Biology, Faculty of Science, Chulalongkorn University, Phayathai Road, Patumwan, Bangkok 10330, Thailand Chulalongkorn University Bangkok Thailand; 4 Royal Belgian Institute of Natural Sciences, Vautierstraat 29, B-1000 Brussels, Belgium Royal Belgian Institute of Natural Sciences Brussels Belgium; 5 Evolutionary Ecology Group, Department of Biology, University of Antwerp, Universiteitsplein 1, B-2610 Antwerp, Belgium University of Antwerp Antwerp Belgium; 6 Department of Biology, Faculty of Science, Mahasarakham University, Maha Sarakham 44150, Thailand Mahasarakham University Maha Sarakham Thailand

**Keywords:** Biodiversity, limestone karst, soil fauna, Southeast Asia, taxonomy

## Abstract

A new species of the giant pill millipede genus *Sphaerobelum* is described: *Sphaerobelumturcosa***sp. nov.** from the northeastern part of Thailand. Species delimitation is based on morphological characters and COI sequence data. The new species can be clearly discriminated from congeners by its greenish-blue body color, the face mask-like appearance of the thoracic and anal shields jointly when rolled up, and the combination of the following four characters: (1) the coxa of the second leg laterally with a sharp and long process, (2) the tarsi of legs 4–21 with 6–7 ventral spines, (3) the anterior telopods consisting of four conspicuous telopoditomeres, and (4) the immovable, slender (not strongly humped) and distally curved finger of the posterior telopods without a membranous spot. The interspecific COI sequence divergence between the new species and other *Sphaerobelum* species ranges from 17% to 23% (mean 20%). The intergeneric COI sequence divergence between the new species and *Zephronia* species ranges from 18% to 21% (mean 20%). The relationships among *Sphaerobelum* and *Zephronia* species based on the COI sequence data were not resolved in this study. *Sphaerobelumturcosa***sp. nov.** is restricted to limestone habitat in Loei province and is probably endemic for the Thai fauna.

## ﻿Introduction

Limestone karsts in Southeast Asia are referred to as ‘arks of biodiversity’ and as such are a priority for biodiversity conservation (Clements et al. 2006). Karst hills usually are relatively small, scattered and isolated, but nonetheless may support large numbers of endemic organisms (Schilthuizen 2011; Tolentino et al. 2020), including a wide variety of millipede species (Golovatch 2015; Liu et al. 2017; Liu and Wynne 2019).

The recent checklist of the millipedes (Diplopoda) of Thailand revealed 263 species. Of these, 222 species (84%) occur only in Thailand and are thus supposed to be endemic (Enghoff 2005; Likhitrakarn et al. 2023). The majority of Thai endemic millipede taxa exclusively inhabit limestone areas and habitats, e.g., *Plusioglyphiulus* Silvestri, 1923 (Golovatch et al. 2011a), *Glyphiulus* Gervais, 1847 (Golovatch et al. 2011b), *Orthomorpha* Bollman, 1893 (Likhitrakarn et al. 2011), *Desmoxytes* Chamberlin, 1923 (Srisonchai et al. 2018) and *Coxobolellus*Pimvichai et al., 2020 (Pimvichai et al. 2020).

Recent fieldwork conducted in the impressive geological landscape at Phu Pha Lom Forest Park, Loei Province in the northeastern part of Thailand, has unveiled remarkable greenish-blue specimens of a giant pill-millipede which clearly belongs to the family Zephroniidae in the order Sphaerotheriida. Recently, the species diversity of this family in Thailand has gained considerable attention (Likhitrakarn et al. 2021; Rosenmejer et al. 2021; Srisonchai et al. 2021; Bhansali and Wesener 2022). The updated records for Zephroniidae in Thailand contains only two genera with a total of 12 valid species (see Likhitrakarn et al. 2023). The number of species, however, is still lower than in some surrounding countries, e.g., Laos, Vietnam and India (Wesener 2019; Semenyuk et al. 2020).

The external characteristics place the specimens within the genus *Sphaerobelum* and the present contribution aims at providing the formal description of this new *Sphaerobelum* species based on morphological and DNA data.

## ﻿Materials and methods

### ﻿Sample collections

Specimens were hand-collected from limestone habitats in Phu Pha Lom Forest Park, Loei Province, Thailand, by visual spotting on open surfaces in daylight. Photographs of live animals were taken with a Canon 70D digital camera with a Canon EF-S 60 mm f/2.8 Macro USM lens. The specimens were euthanized based on AVMA guidelines for the euthanasia of animals (American Veterinary Medical Association 2020), and then preserved in 75% ethanol for morphological study and 95% ethanol for DNA sequence analysis.

The collecting sites were located by GPS using a Garmin GPSMAP 60 CSx, and all coordinates and elevations were rechecked with Google Earth. The background of the distribution map was downloaded from Elastic Terrain Map (http://elasticterrain.xyz/) (Willett et al. 2015) and the figure was composed using Adobe Photoshop CS6.

This research was conducted under the approval of the Animal Care and Use Committee (Protocol Reviews No. IACUC-KKU-136/64 from Khon Kaen University) and No. 1723018 from Chulalongkorn University.

### ﻿Morphological study

The specimens were examined and measured under a Nikon SMZ 745T trinocular stereo microscope, equipped with a Canon EOS 5DS R digital SLR camera. For scanning electron microscopy (SEM), the specimens were photographed with a JEOL, JSM-5410 LV microscope using gold-coated samples. Line drawings were based on photographs taken under the stereo microscope equipped with a digital SLR camera. All final images were processed and edited with Adobe Photoshop CS6.

The terminology of morphological descriptions follows Wesener and Sierwald (2005), Wongthamwanich et al. (2012), Wesener (2016, 2019) and Semenyuk et al. (2018, 2020).

The holotypes, as well as most of the paratypes are housed in the Museum of Zoology, Chulalongkorn University (**CUMZ**), Bangkok, Thailand.

The following abbreviations are used in the figures: **Cx** = coxa, **cp** = cuticular impression, **ia** = inner area, **ma** = middle area, **o** = operculum of vulva, **oa** = outer area, **pm** = posterior margin, **Pre** = prefemur, **St-Pl** = stigmatic plate, **Syn-Cx** = syncoxite.

### ﻿DNA extraction and phylogenetic study

Total genomic DNA was extracted from legs using the NucleoSpin Tissue kit following the manufacturer’s instructions. PCR amplifications and sequencing of the standard mitochondrial COI DNA barcoding fragment (Hebert et al. 2003) were done as described by Pimvichai et al. (2020). The COI fragment was amplified with the primers LCO-1490 and HCO-2198 (Folmer et al. 1994). The new COI nucleotide sequences have been deposited in GenBank under accession numbers OR530087−OR530089. Sample data and voucher codes are provided in Table 1.

**Table 1.** Specimens from which the COI gene fragment was analysed. CUMZ (Museum of Zoology, Chulalongkorn University, Bangkok, Thailand); MHNG (Muséum d’Histoire Naturelle de la Ville de Genève, Geneva, Switzerland); MS (Tokyo Metropolitan University Collection, Tokyo, Japan); NHMD (Natural History Museum of Denmark); SCAU (South China Agricultural University, Guangdong, China); SMF (Senckenberg Museum Frankfurt, Germany); ZFMK (Zoological Research Museum Koenig, Bonn, Germany); ZMUC (Zoologisk Museum, University of Copenhagen, Denmark). Abbreviations after species names refer to the isolate of each sequence. GenBank accession numbers are indicated for each species.

Species	Voucher code	COI accession numbers	Locality	References
**Order Sphaerotheriida**				
**Family Zephroniidae Gray, 1843**				
**Genus *Sphaerobelum* Verhoeff, 1924**				
*S.aesculus*	NHMD 621694	MW898738	Thailand, Nakhon Si Thammarat Province, Khao Luang NP	Rosenmejer et al. 2021
*S.benquii*	SCAU MMY01	OP339792	China, Guizhou, Tongren City, Jiangkou County, Guanhe Town, Guanhe Village, Maomaoyan	Zhao et al. 2022
*S.bolavensis*	MHNG LT-10/24	MK330982	Laos, Champasak Province, Bolaven Plateau, 3 km S of Ban Nong Luang, Tad Kameud	Wesener 2019
*S.denticulatum*	MHNG	MK330984	Laos, Oudomxai Province, ca 3 km E of Tad Lak 11, SE of Oudomxai city	Wesener 2019
*S.huzhengkuni*	SCAU SP03	MT657328	China, Guizhou Province, Tongren City, Fanjingshan National Nature Reserve	Zhao et al. 2020
*S.lachneeis*	MHNG	MK330983	Laos, Oudomxai Province, ca 3 km E of Tad Lak 11, SE of Oudomxai city	Wesener 2019
*S.laoticum*	SMF	MK330975	Laos, Vientiane Province, Vang Vieng	Wesener 2019
*S.meridionalis*	MHNG 4B-2	OM509648	Thailand, Yala Province, Bannang Sata District, Bang Lang National Park, near Than To Waterfall	Bhansali and Wesener 2022
*S.nigrum*	SMF	MK330976	Laos, Champasak Province, Muang Bachieng, Ban Lak 35, Tad Etu	Wesener 2019
*S.peterjaegeri*	SMF SD553	MK330972	Laos, Luang Prabang Province, SE Luang Prabang, Nam Khan, Ban Pak Bak, Houay Kho	Wesener 2019
*S.phouloei*	ZMUC00040257	MK330974	Laos, Houaphan Province, Phou Loei	Wesener 2019
*S.schwendingeri*	MHNG LT 10/03	MK330978	Laos, Vientiane Province, trail to Tham Pou Kham, W. of Vang Vieng	Wesener 2019
*Sphaerobelum* sp. L07	ZMUC00040261	MK330979	Laos, Khammouane Province, Ban Khounkham [Khun Kham] (Nahin)	Wesener 2019
*Sphaerobelum* sp. L10	SMF	MK330980	Laos, Vientiane Province, Vang Vieng, W. of Nam Song, Tham Nam Or Khem	Wesener 2019
*S.spinatum*	ZMUC00040258	MK330973	Laos, Vientiane Province, Phou Khao Khouay	Wesener 2019
*S.truncatum*	FMNH-INS 0000 072 674	JN885184	Thailand, Nan Province, Song Khwae District, Na Rai Luang Subdistrict, Pang Hi Village	Wongthamwanich et al. 2012
*S.tujiaphilum*	SCAU SD02	OP339783	China, Guizhou, Tongren City, Jiangkou County, Guanhe Town, Sidu Village	Zhao et al. 2022
*S.turcosa* sp. nov. SPPL1	CUMZ-Zeph0012	OR530087	Thailand, Loei Province, Mueang Loei District, Phu Pha Lom Forest Park	This study
*S.turcosa* sp. nov. SPPL2	CUMZ-Zeph0012	OR530088	Thailand, Loei Province, Mueang Loei District, Phu Pha Lom Forest Park	This study
**Genus *Zephronia* Gray, 1832**				
*Z.dawydoffi*	ZFMK Myr4504	MK330971	N/A	Wesener 2019
*Z.lannaensis*	ZFMK MYR4911	OM509631	Thailand, Chiang Mai Province, Mae Rim District, Mae Sa Valley	Bhansali and Wesener 2022
*Z.laotica*	ZFMK Myr3502	MK330977	Laos, Champasak Province, east of Mekong, Garden of Erawan Riverside Hotel	Wesener 2019
*Z.ovalis*	ZFMK Myr 0832	JX486068	Vietnam, Dong Nai Province, Cat Tien National Park	Golovatch et al. 2012
*Z.panhai*	ZFMK MYR8116	OM509645	Thailand, Ratchaburi Province, Ratchaburi and Photharam District, 18–20 km WNW of Ratchaburi	Bhansali and Wesener 2022
*Z.phrain*	MYR3500	OM509635	Thailand, Chiang Mai Province, Chiang Mai District, Doi Suthep, behind tourist market	Bhansali and Wesener 2022
*Zephroniasiamensis*	CUMZ	OR530089	Thailand, Chonburi Province, Sichang District, Koh Sichang	This study
*Zephronia* sp.	NHMDK K45	MW898741	Thailand, Prachuap Khiri Khan Province, Mueang district, Aow Noi Temple	Rosenmejer et al. 2021
*Zephronia* sp. 1	ZFMK MYR8787	MW898740	Thailand, Nakhon Si Thammarat Province, Sichon District, Khao Lark Waterfall	Rosenmejer et al. 2021
*Zephronia* sp. 2	NHMD K56x9	OM509650	Thailand, Kanchanaburi Province, Si Sawat District, 50 km W of Kanchanaburi, Erawan Waterfall	Bhansali and Wesener 2022
**Order Glomerida**				
**Family Glomeridae Leach, 1815**				
**Genus *Glomeris* Latreille, 1802**				
*G.marginata*	ZFMK18996	MG931021	Luxemburg, Schengen	Reip and Wesener 2018
**Genus *Hyleoglomeris* Verhoeff, 1910**				
*H.japonica*	MS20210617-02	LC713423	Japan, Kanagawa Prefecture, Fujisawa-shi, Enoshima Island	Kuroda et al. 2022

The COI data included 31 specimens, representing 19 specimens of the genus *Sphaerobelum* and 10 specimens of the genus *Zephronia* (Table 1). Two species of the order Glomerida, viz. *Glomerismarginata* (Villers, 1789) and *Hyleoglomerisjaponica* Verhoeff, 1910 were used as the outgroups.

CodonCode Aligner (ver. 4.0.4, CodonCode Corporation) was used to assemble the forward and reverse sequences and to check for errors and ambiguities. All sequences were checked with the Basic Local Alignment Search Tool (BLAST) provided by NCBI and compared with reference sequences in GenBank. Sequence alignment (660 bp) was done with MUSCLE (ver. 3.6, see http://www.drive5.com/ muscle; Edgar 2004). MEGA11 (ver. 11.0.10, see http://www.megasoftware.net; Tamura et al. 2021) was used to (1) check for stop codons, (2) translate sequences into amino acids, and (3) calculate uncorrected pairwise *p*-distances among sequences. Pairwise deletion of missing data was applied and all positions containing ‘N’s were omitted for each sequenced pair in the analysis.

The best-fit substitution model was implemented using JModelTest2 on XSDXE 2.1.6 (Darriba et al. 2012) through CIPRES Gateway. A phylogenetic tree was constructed using maximum likelihood (ML). The shape parameter of the gamma distribution, based on 16 rate categories, was estimated using maximum likelihood analysis. ML trees were inferred with RAxML (ver. 8.2.12, see http://www.phylo.org/index.php/tools/raxmlhpc2_tgb.html; Stamatakis 2014) through the CIPRES Science Gateway (Miller et al. 2010) using a GTR+G substitution model and 1000 bootstrap replicates to assess branch support.

## ﻿Results

### ﻿COI sequence data

Uncorrected *p*-distances between the sequences range from 0.00 to 0.32 (Table 2). The mean interspecific sequence divergence within *Sphaerobelum* was 0.19 (range: 0.10–0.24). The mean sequence divergence between *S.turcosa* sp. nov. and other *Sphaerobelum* species was 0.20 (range: 0.17–0.23). The mean interspecific sequence divergence within *Zephronia* was 0.19 (range: 0.12–0.23). The mean sequence divergence between *S.turcosa* sp. nov. and *Zephronia* was 0.20 (range: 0.18–0.21). The mean sequence divergence between *Sphaerobelum* and *Zephronia* was 0.21 (range: 0.17–0.25).

**Table 2.** Estimates of cytochrome *c* oxidase I (COI) sequence divergences (uncorrected *p*-distances) within and among Zephroniidae species and related taxa (rounded to two decimal places).

		1	2	3	4	5	6	7	8	9	10	11	12	13	14	15	16	17	18	19	20	21	22	23	24	25	26	27	28	29	30	31
1	*Sphaerobelumbenquii*																															
2	*Sphaerobelumbolavensis*	0.21																														
3	*Sphaerobelumdenticulatum*	0.20	0.20																													
4	*Sphaerobelumhuzhengkuni*	0.18	0.22	0.18																												
5	*Sphaerobelumlachneeis*	0.23	0.20	0.21	0.21																											
6	*Sphaerobelumlaoticum*	0.19	0.21	0.18	0.16	0.21																										
7	*Sphaerobelumnigrum*	0.19	0.19	0.20	0.19	0.21	0.19																									
8	*Sphaerobelumpeterjaegeri*	0.19	0.16	0.19	0.18	0.21	0.19	0.19																								
9	*Sphaerobelumphouloei*	0.20	0.18	0.17	0.17	0.21	0.18	0.19	0.18																							
10	*Sphaerobelumschwendingeri*	0.19	0.18	0.18	0.17	0.21	0.14	0.20	0.18	0.17																						
11	*Sphaerobelum* sp. L07	0.21	0.21	0.22	0.20	0.24	0.21	0.21	0.19	0.20	0.20																					
12	*Sphaerobelum* sp. L10	0.19	0.19	0.20	0.21	0.22	0.16	0.17	0.18	0.18	0.16	0.19																				
13	*Sphaerobelumaesculus*	0.20	0.20	0.21	0.19	0.24	0.21	0.22	0.19	0.21	0.20	0.22	0.20																			
14	*Sphaerobelummeridionalis*	0.19	0.20	0.20	0.18	0.22	0.18	0.20	0.18	0.17	0.18	0.21	0.19	0.17																		
15	*Sphaerobelumspinatum*	0.22	0.18	0.20	0.20	0.22	0.20	0.21	0.19	0.18	0.20	0.20	0.20	0.24	0.22																	
16	*Sphaerobelumtruncatum*	0.21	0.18	0.20	0.19	0.21	0.18	0.20	0.10	0.18	0.19	0.21	0.19	0.20	0.19	0.21																
17	*Sphaerobelumtujiaphilum*	0.20	0.21	0.18	0.12	0.21	0.18	0.20	0.19	0.17	0.17	0.21	0.21	0.21	0.17	0.19	0.20															
18	*Sphaerobelumturcosa* sp. nov. SPPL1	0.21	0.19	0.20	0.18	0.23	0.19	0.20	0.18	0.17	0.18	0.21	0.20	0.21	0.20	0.21	0.18	0.18														
19	*Sphaerobelumturcosa* sp. nov. SPPL2	0.20	0.19	0.20	0.18	0.23	0.18	0.20	0.18	0.17	0.18	0.21	0.19	0.21	0.20	0.21	0.18	0.18	0.00													
20	*Zephroniadawydoffi*	0.21	0.22	0.22	0.18	0.23	0.20	0.19	0.20	0.19	0.21	0.20	0.21	0.20	0.19	0.21	0.20	0.20	0.18	0.18												
21	*Zephronialannaensis*	0.22	0.19	0.22	0.20	0.22	0.22	0.20	0.21	0.21	0.21	0.22	0.22	0.22	0.20	0.22	0.20	0.20	0.20	0.20	0.17											
22	*Zephronialaotica*	0.25	0.23	0.22	0.20	0.24	0.22	0.19	0.21	0.21	0.23	0.23	0.24	0.21	0.20	0.23	0.22	0.21	0.20	0.20	0.16	0.18										
23	*Zephroniaovalis*	0.22	0.21	0.24	0.21	0.23	0.23	0.20	0.20	0.22	0.23	0.25	0.22	0.23	0.23	0.22	0.21	0.22	0.20	0.20	0.15	0.20	0.16									
24	*Zephroniapanhai*	0.20	0.20	0.23	0.21	0.23	0.21	0.20	0.21	0.22	0.21	0.23	0.22	0.22	0.21	0.21	0.21	0.19	0.19	0.19	0.18	0.15	0.20	0.19								
25	*Zephroniaphrain*	0.22	0.21	0.24	0.19	0.24	0.20	0.17	0.20	0.20	0.20	0.22	0.21	0.21	0.21	0.22	0.21	0.20	0.19	0.19	0.19	0.22	0.19	0.20	0.21							
26	*Zephroniasiamensis*	0.21	0.21	0.21	0.19	0.24	0.22	0.19	0.19	0.19	0.23	0.21	0.21	0.19	0.19	0.21	0.20	0.20	0.19	0.19	0.10	0.19	0.14	0.15	0.19	0.19						
27	*Zephronia* sp.	0.23	0.21	0.23	0.21	0.24	0.19	0.21	0.19	0.20	0.20	0.20	0.19	0.20	0.20	0.21	0.21	0.19	0.20	0.20	0.20	0.23	0.22	0.23	0.21	0.20	0.21					
28	*Zephronia* sp. 1	0.23	0.20	0.24	0.21	0.24	0.23	0.20	0.20	0.21	0.22	0.22	0.22	0.20	0.21	0.22	0.21	0.21	0.21	0.21	0.22	0.22	0.21	0.22	0.20	0.21	0.21	0.17				
29	*Zephronia* sp. 2	0.19	0.20	0.20	0.19	0.20	0.19	0.19	0.18	0.21	0.21	0.22	0.20	0.19	0.18	0.21	0.18	0.18	0.19	0.19	0.17	0.13	0.18	0.18	0.12	0.21	0.17	0.19	0.21			
30	*Glomerismarginata*	0.29	0.29	0.29	0.27	0.33	0.28	0.29	0.28	0.27	0.30	0.29	0.29	0.28	0.30	0.28	0.29	0.28	0.29	0.29	0.30	0.30	0.31	0.30	0.30	0.29	0.29	0.27	0.29	0.29		
31	*Hyleoglomerisjaponica*	0.29	0.29	0.30	0.29	0.33	0.28	0.30	0.30	0.27	0.28	0.30	0.28	0.29	0.32	0.30	0.30	0.29	0.30	0.30	0.30	0.31	0.31	0.32	0.30	0.30	0.31	0.28	0.31	0.31	0.15	

**Figure 1.**
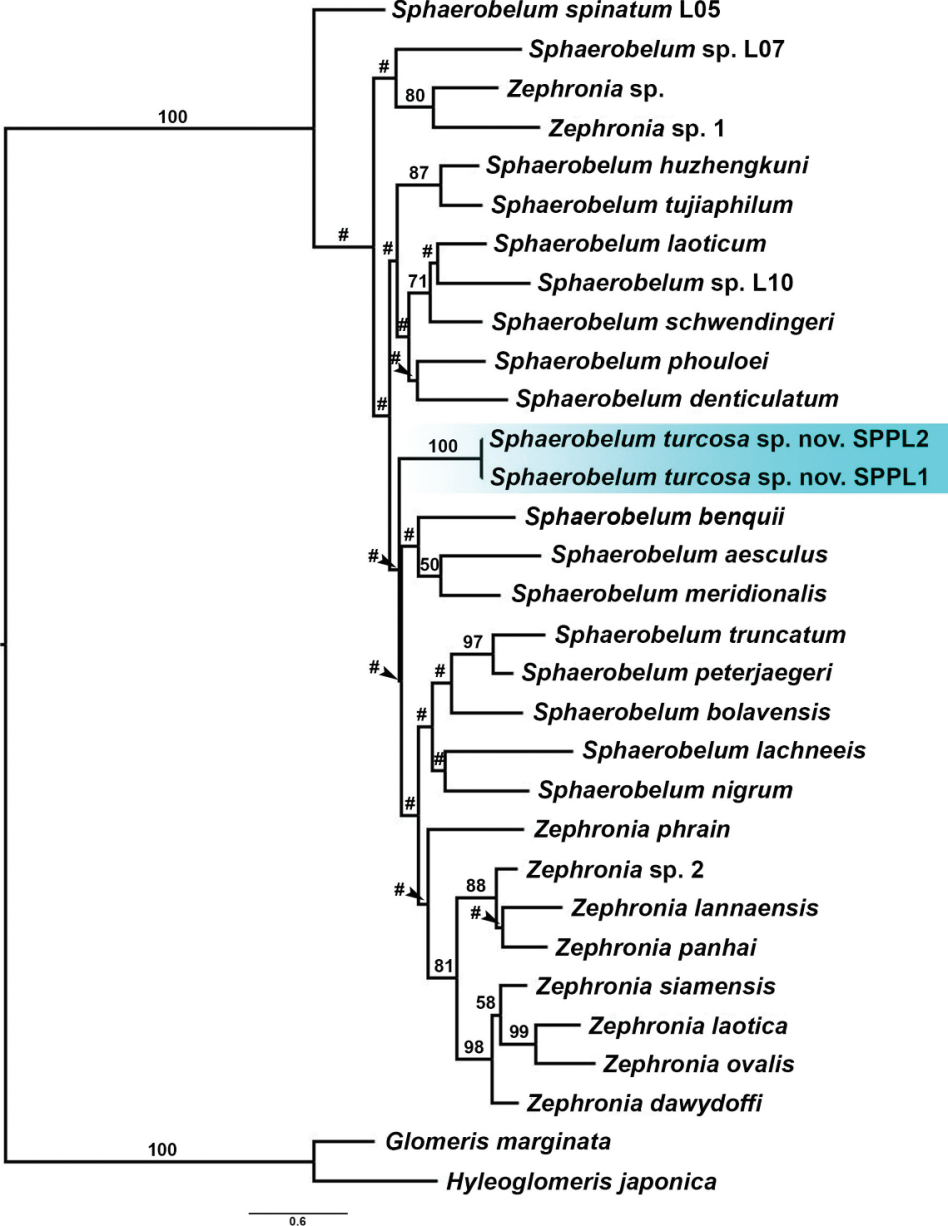
The COI gene tree based on maximum likelihood analysis of cytochrome *c* oxidase I (COI) (660 bp). Numbers at nodes indicate branch support based on bootstrapping. Scale bar: 0.6 substitutions per site. # marks branches with <50% bootstrap support. The colored area marks *Sphaerobelumturcosa* sp. nov.

In the phylogenetic tree based on the COI gene (Fig. 1), the clade of Zephroniidae (*Sphaerobelum* + *Zephronia*) is well supported by ML (bootstrap support = 100), but the relationships among *Sphaerobelum* and *Zephronia* species could not be resolved.

### ﻿Taxonomy

**Family Zephroniidae Gray, 1843**

**Subfamily Zephroniinae Gray, 1843**

**Tribe Zephroniini Gray, 1843**

**Genus *Sphaerobelum* Verhoeff, 1924**

#### ﻿ Sphaerobelum turcosa

Taxon classificationAnimaliaSphaerotheriidaZephroniidae

Srisonchai & Pimvichai sp. nov.

A05DED66-F292-50F3-9B76-469CE1B5D43B

https://zoobank.org/518D6D7F-12F1-4703-97A3-006C7663CE20

Figs 2 , 3 , 4 , 5

##### Materials examined.

***Holotype*** ♂ (CUMZ-Zeph0011), Thailand, Loei Province, Mueang Loei District, Phu Pha Lom Forest Park, 383 m a.s.l., 17°33'16"N, 101°52'06"E, 10/07/2014, leg. R. Srisonchai and C. Sutcharit.

***Paratypes*.** 5 ♂, 2 ♀ (CUMZ-Zeph0012), same data as holotype. 1 ♂, 2 ♀ (CUMZ-Zeph0012), same data as holotype, 01/08/2020 and 25/09/2021, leg. P. Pimvichai, P. Prasankok and S. Saratan. 1 ♂, 2 ♀ (CUMZ-Zeph0012), same District, Wat Phu Pha Lom, 265 m a.s.l., 17°33'16"N, 101°52'04"E, 14/05/2008, leg. C. Sutcharit.

##### Etymology.

The specific name is a Latin adjective, meaning ‘turquoise, greenish-blue mineral,’ and refers to the general body color of living specimens.

##### Diagnosis.

Coxal process on leg 2 sharply projecting, tarsi of legs 4–21 with 4/5/6/7/8 ventral spines. Similar in these respects to *S.lachneeis*, *S.schwendingeri* and *S.laoticum*, but *S.turcosa* sp. nov. differs from them by the combination of several characters, viz. body yellow contrasting to dominant greenish-blue color (vs. dark green/black); mesal margin of femur with teeth (vs. without teeth); vulva board and large, covering mesal 2/3 of coxa (vs. narrower, covering mesal 1/3 or half of coxa); anterior telopod consisting of 4 conspicuous telopoditomeres (vs. 3 telopoditomeres); immovable fingers of posterior telopod slender (vs. strongly humped and swollen).

##### Description of the new species.

***Measurements***: Male Holotype. Body length ca 18.5 mm. Width, of thoracic shield = 9.5 mm, of tergite 8 = 10.3 mm (= broadest). Height, of thoracic shield = 5.1 mm, of tergite 7 = 5.3 mm (= highest). Male: body length = 15.2–18.4 mm. Width, of thoracic shield = 7.9–9.0 mm, of tergite 8 = 8.7–9.4 mm. Height, of thoracic shield = 4.8–5.6 mm, of tergite 7, 5.2–6.2 mm. Female: body length = 20.6–24.5 mm. Width, of thoracic shield = 6.3–10.6 mm, of tergite 8 = 7.2–11.6 mm. Height, of thoracic shield = 5.4–6.0 mm, of tergite 7, 5.7–6.7 mm.

**Figure 2.**
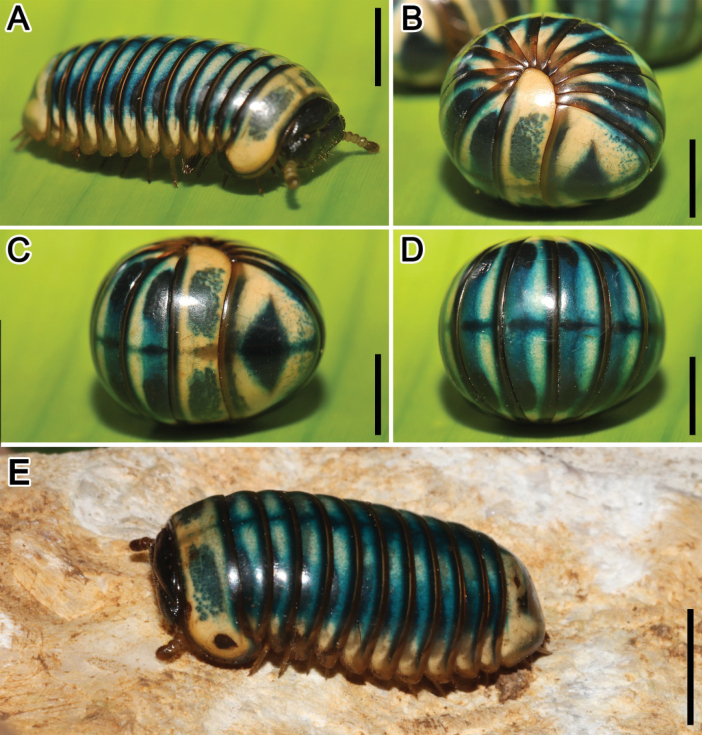
Habitus, live coloration. *Sphaerobelumturcosa* sp. nov., ♀ paratype **A, E** sublateral views **B–D** enrolled, sublateral, lateral, dorsal views, respectively. Scale bars: 5 mm.

***Coloration***: Live animals yellow with contrasting greenish-blue anterior margins of tergites and darker blue dorsal axial stripe (Fig. 2A–D), thoracic shield with a large, paramedian, greenish-blue band at middle (Fig. 2A–C), anal shield with a large, greenish-blue diamond at axial line (Fig. 2B, C), head, collum and groove of thoracic blackish to dark blue (Fig. 2A), legs, antennae, paratergite depressions and venter light brown to brown, venter brown to yellow brownish; coloration in alcohol, after more than 10 years of preservation, faded to light yellow with contrasting greenish-blue anterior margins of tergites, head, collum and groove of thoracic dark blue to blue, legs, antennae, paratergite depressions and venter brown to yellowish.

***Head***: trapezoid, anterior part of the head with many long setae, posterior part densely dimpled; anterior margin of labrum with a single tooth. Eyes with 37–63 ocelli (male) or 55–67 (female). Aberrant ocellus located inside antennal groove.

***Antennae***: short, with rounded joints, extending posteriorly to leg-pair 3. Lengths of antennomeres: 2<3=4<1<5<6. All antennomeres densely pubescent, sensilla basiconica surrounding apical disc. Last antennomere thickened, apically widened and well rounded (Fig. 2A). Apical disc with 22–42 apical cones (male) or 21–29 (female). No sclerotized crest/ridge between antenna socket and eye field. Tömösváry organ located between eye field and antenna socket, next to, but separated from eye field.

***Gnathochilarium***: Structure typical of the Sphaerotheriida. Palpi with sensory cones arranged in clusters.

***Mandibles***: not dissected.

***Collum***: with glabrous surface, except for anterior and posterior margin with a few isolated and long setae.

**Figure 3.**
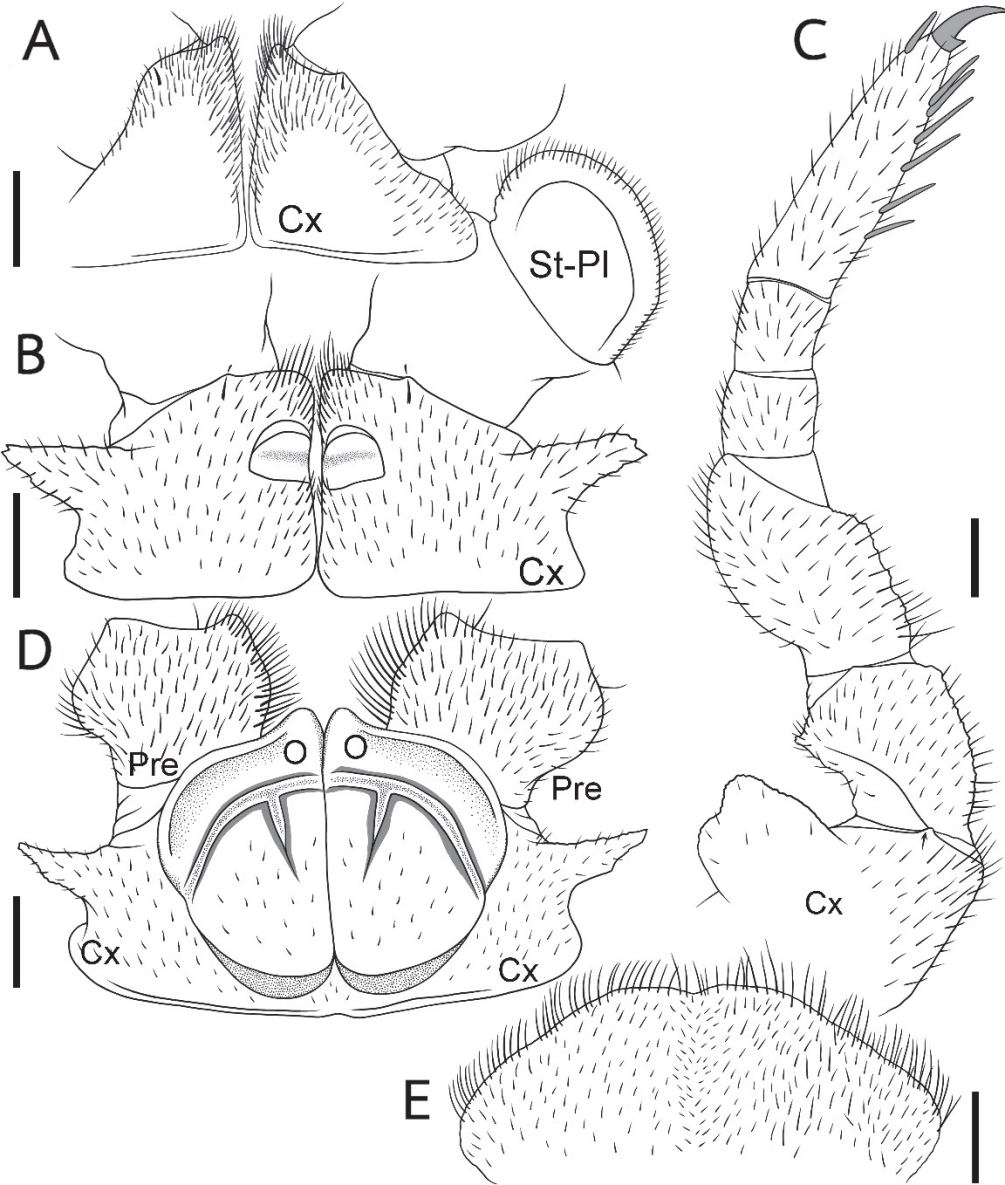
*Sphaerobelumturcosa* sp. nov. **A–C** ♂ holotype **D, E** ♀ paratype **A** first left coxa with stigmatic plate **B** coxa of second leg with gonopore **C** ninth right leg **D** coxa and prefemur of second leg with vulva **E** subanal plate. Scale bars: 0.5 mm.

***Stigmatic plates***: first stigmatic plate rounded, apex well-rounded, slightly curved towards coxa (Fig. 3A).

***Laterotergites***: laterotergite 1 strongly projecting into a sharp tip. Laterotergite 2 with a broad, stout projection and a deep notch at lateral margin, like following laterotergites.

***Following tergites***: surface glabrous, shining, except the groove of paratergite with tiny setae. Tips of paratergites of midbody tergites projecting posteriorly.

***Thoracic shield***: surface glabrous as in tergites. Shallow grooves with few setae, surface glabrous, no keels.

***Endotergum***: Posterior margin (pm) flat, regular (Fig. 5A). Outer area (oa) without setae. Middle area (ma) with a single row, conspicuous, elliptical cuticular impressions (cp); distance between impressions as long as individual diameter (Fig. 5B). Bristles arranged in two rows, tip of the longest bristles not extended beyond posterior margin or not reaching to posterior margin (Fig. 5A). Inner area (ia) without tubercles or setae, but with small pits (Fig. 5D).

***Anal shield***: slightly sexually dimorphic, in female large and well-rounded (Fig. 2B), in male slightly more rectangular, in both sexes glabrous. Surface similar to that of tergites. Underside with a single, very short, black locking carina, six times shorter than width of last laterotergite.

***Legs***: leg-pair 1 with 1 or 2 ventral spines, leg-pair 2 with 3 or 4, leg-pair 3 with 5 or 6. First two leg-pairs without an apical spine. Leg pairs 4–21 with 6 or 7 ventral spines and one apical spine. In leg 9, femur 1.6 times, tarsus 3.5 times longer than wide (Fig. 3C). Femur extended mesally into a dentate margin featuring 10–14 teeth. All podomeres densely setose. Coxa with a large and marginally toothed process. Coxal process absent at first leg and sharply projecting at second (Fig. 3B). Prefemur at apical margin with a projection laterally and mesally. Lateral projection triangular and sharply edged, juxtaposed to coxal process (Fig. 3C).

***Female sexual characters***: vulva large, covering 2/3 of coxa, located at mesal margin, extending mesally to anterior third of prefemur (Fig. 3E). Operculum rounded, mesal margin projecting into a well-rounded lobe 1/2 as high as remaining operculum. Subanal plate: large and wide, divided by a suture into two halves. Densely setose (Fig. 3D).

***Male sexual characters***: gonopore large, covered with a single, undivided, triangular, sclerotized plate (Fig. 3B).

**Figure 4.**
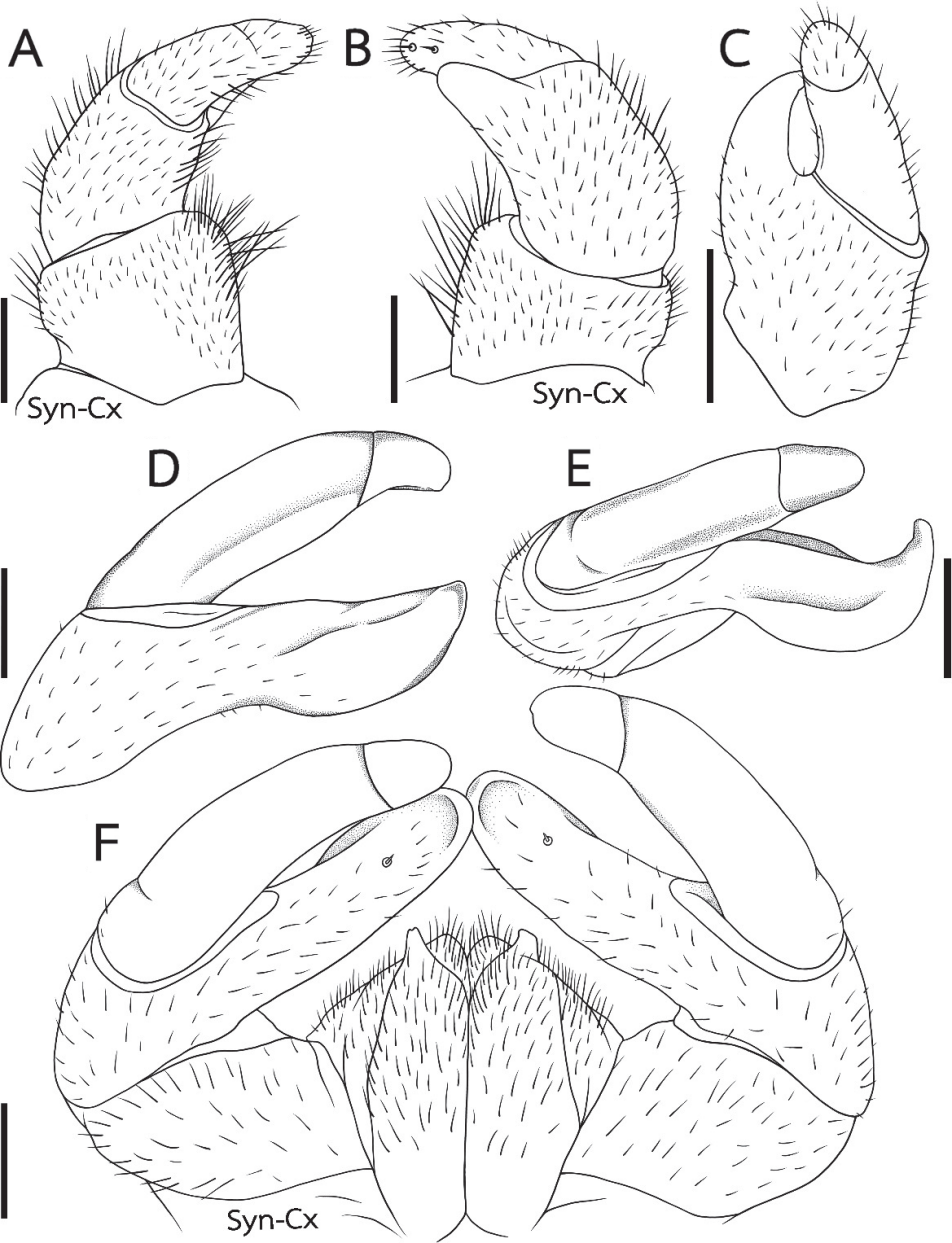
*Sphaerobelumturcosa* sp. nov. **A–C** ♂ holotype **D–F** ♂ paratype **A–C** left anterior telopods, anterior, posterior and lateral views, respectively **D, E** left posterior telopod, posterior, subventral views **F** posterior telopods, anterior view. Scale bars: 0.5 mm.

***Anterior telopods*** (Fig. 4A–C): First podomere rectangular, slightly wider than long. Telopoditomere 2 large, as long as telopoditomere 3. Immovable finger (process of telopoditomere 2) wide, located posteriorly, but partly visible laterally in anterior view, projecting to half of movable finger (telopoditomeres 3 and 4), slightly curved, apically with a rounded tip. Telopoditomeres 3 and 4 divided by a short and weak suture, this suture being almost visible in lateral view (Fig. 4A–C). Telopoditomere 3 large, cylindrical, slender, 1.2 times longer than wide, 2 times longer than telopoditomere 4 (Fig. 4A, C). Telopoditomere 4 short, well-rounded, posterior face with two small, sclerotized spot and triangular spines (Fig. 4B). All podomeres covered with long setae.

**Figure 5.**
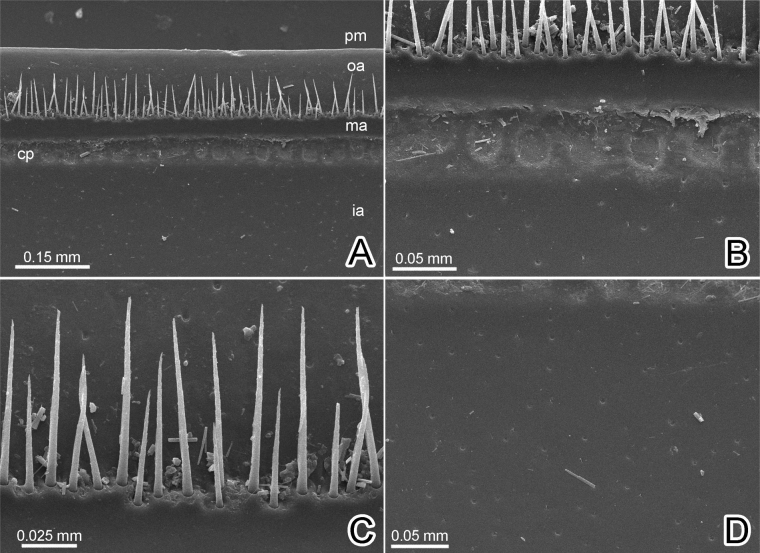
*Sphaerobelumturcosa* sp. nov. ♂ paratype, SEM micrographs of the endoterga on the midbody segment. **A** all areas of endotergum, posterior margin (pm), outer area (oa), middle area (ma), cuticular impressions (cp) and inner area (ia) **B** middle area and cuticular impressions **C** bristles **D** inner area.

***Posterior telopods*** (Fig. 4D–F): inner horns with sharp-edged tips, slightly curved caudad. Telopodite consisting of four podomeres. First telopoditomere rectangular, slightly longer than wide. Immovable finger (process of telopoditomere 2) as long as movable finger, consisting of telopoditomeres 3 and 4. Immovable finger wide, 2.5 times longer than wide, with a characteristic, distally swollen, clearly rounded apically, apex only slightly wider than base; tip strongly curved when seen in dorsolateral view. Immovable finger in anterior view with a small spine, at middle with sclerotized spot. Telopoditomere 3 long and slender, 2.5 times longer than wide, with a membranous lobe at a excavate inner margin. Telopoditomere 4 very short and slender, only 4.5 times shorter than telopoditomere 3, 1.5 times longer than wide, slightly tapering apically. Telopoditomere 4 with one small, weak spine at margin towards immovable finger. Telopoditomere 1 and 2 at both sides covered by setae. Telopoditomere 3 only basally in anterior view with setae, remaining part, as well as telopoditomere 4 glabrous.

##### Distribution and habitat.

**Figure 6.**
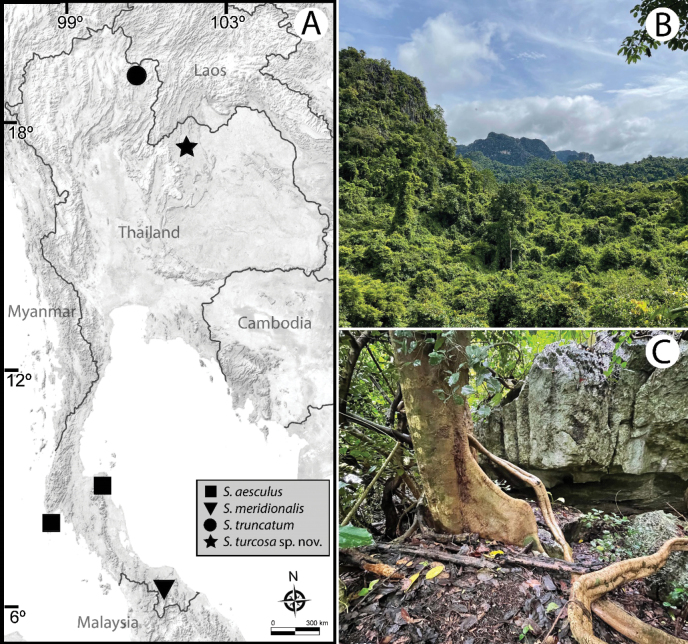
Distribution and type locality of *Sphaerobelumturcosa* sp. nov. **A** distribution map of *Sphaerobelum* species in Thailand **B, C** limestone habitat at the type locality.

Currently known only from the type locality. All specimens were crawling openly on the bottom of several holes in humid rocks (Fig. 6). The stark bright color invited collectors to pick them up.

### ﻿Key to species of the genus *Sphaerobelum* in Thailand (4 species)

**Table:** 

1	Legs 4–21 usually with one apical spine. Posterior telopods: immovable finger of telopoditomere 2 without membranous spot	**2**
–	Legs 4–21 usually with three apical spines. Posterior telopods: immovable finger of telopoditomere 2 with membranous spot, visible in posterior view	**3**
2	Greenish-blue body color. Male body length 15.2–18.4 mm. Endotergum with regular flat margin (Fig. 5). Leg 3 without apical spine. Coxa of second leg laterally with a sharp and long process (Fig. 3B, D). Posterior telopods: distal part of immovable finger of telopoditomere 2 moderately enlarged, tip strongly curved	***S.turcosa* sp. nov.**
–	Brown body color. Male body length 18.6–24.0 mm. Endotergum with ‘rectangle-wavy’ margin. Leg 3 with one apical spine. Coxa of second leg laterally without a sharp and long process. Posterior telopods: distal part of immovable finger of telopoditomere 2 strongly enlarged, tip not curved	***S.truncatum* Wongthamwanich, 2012**
3	Endotergum with two rows of bristles. First coxae with mesal process. Prefemur of midbody legs with mesal process. Telopoditomere 3 and 4 of anterior telopods clearly separated	***S.meridionalis* Bhansali & Wesener, 2022**
–	Endotergum with one row of bristles. First coxae without mesal process. Prefemur of midbody legs without mesal process. Telopoditomere 3 and 4 of anterior telopods incompletely fused	***S.aesculus* Rosenmejer & Wesener, 2021**

## ﻿Discussion

The new species can be distinguished from congeners by its greenish-blue color in combination with (1) a protruding coxal process, (2) a slender shape of the immovable finger, and (3) the shape of the posterior telopod. Yet, on the basis of morphology, little can be said about the relationships with other putatively closely related species. The overlapping COI *p*-distances and the phylogenetic tree were unable to resolve the relationships both within and among the genera *Sphaerobelum* and *Zephronia*. A similar unresolved tree was reported for the genus *Sphaerobelum* by Wesener (2019). Thus, the phylogenetic relationships of the *Sphaerobelum* and *Zephronia* species remain unclear and need further investigations.

The new species exhibits a striking greenish-blue coloration, by which it joins other brightly colored millipedes such as the genus *Apheloria* (*A.polychroma*) and the genus *Desmoxytes* (*D.purpurosea*, *D.rubra* and *D.aurata*). Species of this latter genus are not only very colorful, but they also have long spine-like paraterga. These characteristics are probably aposematic (Enghoff et al. 2007; Srisonchai et al. 2018).

The occurrence of the new species in Phu Pha Lom Forest Park is possibly correlated with the type of habitat/microhabitats. Due to its heterogenous topologies, its strongly irregular geomorphology and the good drainage of the limestone substrate, the type locality of *S.turcosa* sp. nov. probably provides suitable conditions to harbour a rich soil fauna.

With the discovery of *S.turcosa* sp. nov., the number of *Sphaerobelum* species in Thailand increases to four, which is still less than the number of *Sphaerobelum* species in Laos (10 species) and Vietnam (6 species) (Wesener 2019; Semenyuk et al. 2020), but more than in Cambodia and Myanmar, where hitherto no *Sphaerobelum* species have been reported (Likhitrakarn et al. 2014, 2015).

## Supplementary Material

XML Treatment for Sphaerobelum turcosa

## ﻿Additional information

### Author ORCIDs

Ruttapon Srisonchai https://orcid.org/0000-0002-7142-0999

Natdanai Likhitrakarn https://orcid.org/0000-0002-1306-317X

Chirasak Sutcharit https://orcid.org/0000-0001-7670-9540

Thierry Backeljau https://orcid.org/0000-0002-9057-9727

Piyatida Pimvichai https://orcid.org/0000-0001-9765-821X

### Data availability

All of the data that support the findings of this study are available in the main text.
